# A *P. falciparum* NF54 Reporter Line Expressing mCherry-Luciferase in Gametocytes, Sporozoites, and Liver-Stages

**DOI:** 10.3389/fcimb.2019.00096

**Published:** 2019-04-16

**Authors:** Catherin Marin-Mogollon, Ahmed M. Salman, Karin M. J. Koolen, Judith M. Bolscher, Fiona J. A. van Pul, Shinya Miyazaki, Takashi Imai, Ahmad Syibli Othman, Jai Ramesar, Geert-Jan van Gemert, Hans Kroeze, Severine Chevalley-Maurel, Blandine Franke-Fayard, Robert W. Sauerwein, Adrian V. S. Hill, Koen J. Dechering, Chris J. Janse, Shahid M. Khan

**Affiliations:** ^1^Department of Parasitology, Leiden University Medical Center, Leiden, Netherlands; ^2^Nuffield Department of Medicine, The Jenner Institute, University of Oxford, Oxford, United Kingdom; ^3^TropIQ Health Sciences, Nijmegen, Netherlands; ^4^Department of Infectious Diseases and Host Defense, Gunma University Graduate School of Medicine, Maebashi, Japan; ^5^Faculty of Health Sciences, Universiti Sultan Zainal Abidin, Terengganu, Malaysia; ^6^Department of Medical Microbiology, Radboud University Medical Center, Nijmegen, Netherlands

**Keywords:** *Plasmodium falciparum*, transgenes, reporters, mCherry, luciferase, CRISPR/Cas9

## Abstract

Transgenic malaria parasites expressing fluorescent and bioluminescent proteins are valuable tools to interrogate malaria-parasite biology and to evaluate drugs and vaccines. Using CRISPR/Cas9 methodology a transgenic *Plasmodium falciparum* (Pf) NF54 line was generated that expresses a fusion of *mCherry* and *luciferase* genes under the control of the *Pf etramp10*.3 gene promoter (line mCherry-luc@etramp10.3). *Pf etramp10*.3 is related to rodent *Plasmodium uis4* and the *uis4* promoter has been used to drive high transgene expression in rodent parasite sporozoites and liver-stages. We examined transgene expression throughout the complete life cycle and compared this expression to transgenic lines expressing mCherry-luciferase and GFP-luciferase under control of the constitutive *gapdh* and *eef1a* promoters. The *mCherry-luc@etramp10.3* parasites express mCherry in gametocytes, sporozoites, and liver-stages. While no mCherry signal was detected in asexual blood-stage parasites above background levels, luciferase expression was detected in asexual blood-stages, as well as in gametocytes, sporozoites and liver-stages, with the highest levels of reporter expression detected in stage III-V gametocytes and in sporozoites. The expression of mCherry and luciferase in gametocytes and sporozoites makes this transgenic parasite line suitable to use in *in vitro* assays that examine the effect of transmission blocking inhibitors and to analyse gametocyte and sporozoite biology.

## Introduction

Transgenic rodent and human malaria parasites expressing fluorescent and bioluminescent proteins are used extensively to interrogate parasite biology and host-parasite interactions associated with malaria pathology and as tools to evaluate anti-parasite inhibitors and vaccines (Othman et al., 2017). In comparison to transgenic rodent malaria parasites (RMP) only a few *Plasmodium falciparum* (*Pf*) transgenic parasites expressing fluorescent or luminescent proteins are available. Transgenic *Pf* parasites have been used to quantify effects of inhibitors and antibodies either on blood-stage growth *in vitro* using standard growth inhibition assays (Wilson et al., 2010) or on transmission-blocking (TB) activity in the mosquito in high-throughput screens using standard membrane feeding assays (Vos et al., 2015; Lucantoni et al., 2016). For the TB assays transgenic *Pf* (NF54 strain) parasite lines expressing the GFP-luciferase fusion protein under control of either the strong constitutive *Pf hsp70* (Vos et al., 2015) or the gametocyte-specific *pfs16* promoters have been used (Lucantoni et al., 2016). In addition, a transgenic *Pf* NF54 has been described that expresses a GFP-luciferase fusion protein under control of the constitutive *Pf eef1a* promoter (Vaughan et al., 2012). This reporter line has been used to analyze liver infection in immune-deficient humanized mice engrafted with human liver tissue ((Sack et al., 2014; Flannery et al., 2018); (Foquet et al., 2018)).

In multiple RMP transgenic lines the *uis4* gene promoter has been used to drive expression of different transgenes in sporozoites and liver-stages, such as genes encoding mCherry, ovalbumin or human *Plasmodium* proteins (Combe et al., 2009; Panchal et al., 2012; Montagna et al., 2014; Hopp et al., 2015; Longley et al., 2015, 2017; Singer et al., 2015) The *uis4* gene, a member of the small *Plasmodium etramp* gene family, is highly transcribed in sporozoites and liver-stages and encodes a parasitophorous vacuole membrane (PVM) protein that surrounds the parasite in the infected hepatocyte. Although *uis4* transcripts are translationally repressed in sporozoites (Silvie et al., 2014; Silva et al., 2016), transgenes expressed under control of *uis4* regulatory sequences are expressed as protein in sporozoites since *uis4* translational repression in sporozoites is dependent on DNA sequences present within the *uis4* open reading frame (Silvie et al., 2014; Hopp et al., 2015). In this study, we generated a transgenic *Pf* parasite, *mCherry-luc@etramp10.3*, which encodes a fusion of the proteins mCherry and luciferase (mCherry-Luc) expressed under the control of the promoter of *etramp10.3* (PF3D7_1016900). We selected this promoter since *etramp10.3* is related to *uis4* and it also belongs to the *etramp* gene family and both genes have the same syntenic genomic location. It has been previously reported that *etramp10.3* is expressed in *Pf* sporozoites as well as in blood- and liver-stages, where the protein is located at the PVM, similar to the location of UIS4 in liver-stages of rodent malaria parasites (Mackellar et al., 2010). In this study, as a blood-stage reporter control line, we also generated a transgenic *Pf* parasite that expresses mCherry-Luc under the control of the promoter of *gapdh* (PF3D7_1462800), *mCherry-luc@gapdh*. The *gapdh* gene is constitutively expressed in blood-stages (Aurrecoechea et al., 2009) and we recently showed that the *gapdh* promoter drives high GFP expression in both asexual blood-stages and in gametocytes (Mogollon et al., 2016).

We chose to generate mCherry-expressing *Pf* reporter lines as such lines could be used to visualize interactions of *Plasmodium* parasites with host-cells (e.g., cells of the immune system or hepatocytes), which are often labeled with green fluorescent proteins. Moreover, we fused the mCherry gene to firefly luciferase, as luminescence can be used to quantify parasite numbers (e.g., sporozoites and liver-stages) using standardized and sensitive assays (Annoura et al., 2013; Le Bihan et al., 2016; Swann et al., 2016). The mCherry-luciferase expression cassettes were introduced using a CRISPR/Cas9 methodology into the *p47* gene locus, a non-essential locus that has been previously used to introduce transgenes into the *Pf* genome (Talman et al., 2010; Vaughan et al., 2012; Vos et al., 2015; Lucantoni et al., 2016). Reporter gene expression of *mCherry-luc@etramp10.3* line was analyzed throughout the complete parasite life cycle and compared to transgenic lines expressing mCherry-luciferase or GFP-luciferase under control of the constitutive *gapdh* or *eef1a* promoters. We demonstrate mCherry and luciferase expression in *mCherry-luc@etramp10.3* gametocytes, sporozoites and liver-stages.

## Materials and Methods

### *In vitro* Cultivation of *P. falciparum* Blood-Stages

*P. falciparum* (*Pf*) parasites from the NF54 strain were used (Mogollon et al., 2016). Parasites were cultured using standard culture conditions in a semi-automated culture system as described (Mogollon et al., 2016) Fresh human serum and human red blood cells (RBC) were obtained from the Dutch National Blood Bank (Sanquin Amsterdam, the Netherlands; permission granted from donors for the use of blood products for malaria research and microbiology tested for safety). RBC of different donors were pooled every 2 weeks, washed twice in serum free RPMI-1640 and suspended in complete culture medium to 50% hematocrit. Human serum of different donors were pooled every 4–6 months and stored at −20°C until required. *Pf* gametocytes cultures were generated using standard culture conditions with some modifications as described (Mogollon et al., 2016). Briefly, parasites from asexual stage cultures were diluted to a final parasitemia of 0.5% and cultures were followed during 14 days without refreshing RBC. At day 14 the cultures were analyzed for mature, stage V, gametocytes.

In addition, a transgenic *P. falciparum* (NF54) line (*gfp-luc@eef1a*) was used that contains a reporter cassette with a fusion gene of GFP and luciferase (GFP-Luc) under control of *eukaryotic elongation factor 1 alpha* (*eef1a*) promoter (Vaughan et al., 2012).

### Generation of the *mcherry-luc@etramp10.3* and the *mCherry-luc@gapdh* Parasite Lines

To create the *mCherry-luc@etramp* reporter line we used a previously described Cas9 construct (pLf0019), containing the Cas9 expression cassette with a *blasticidin* (BSD) drug-selectable marker cassette (Mogollon et al., 2016) in combination with a sgRNA donor-DNA plasmid (pLf0049). The sgRNA-donor DNA construct (pLf0049) contains a h*dhfr-yfcu* drug-selectable marker (SM) cassette for selection with the drug WR99210. To generate pLf0049, the intermediate plasmid pLf0039 (Supplementary Figure 1) was modified by introducing two homology regions targeting of *p47* (PF3D7_1346800). Homology region 1 (HR1) was amplified using primers P1/P2 and homology region 2 (HR2) with P3/P4 from *Pf* NF54 genomic DNA (see Supplementary Table 1 for primer details). HR1 was cloned in pLf0039 using restriction sites *StuI*I/*Sac*II and HR2 using *Apa*I/*Hind*III. Subsequently, a guide RNA sgRNA targeting the *p47* locus (gRNA019) was selected using the Protospacer software (alphaversion; https://sourceforge.net/projects/ protospacerwb/files/Release/), based on the best off targets hits score throughout the genome given by Protospacer and the total number of mismatches of the sgRNA with respect to the PAM site. A 20 bp guide sgRNA sequence (using the primers P5/P6), flanked on both sides by a 15 bp DNA sequence necessary for In Fusion cloning (HD Cloning Kit; Clontech), was annealed and used to replace the BtgZI adaptor in pLf0039 as previously described (Ghorbal et al., 2014), resulting in pLf0047. The plasmid pLf0047 was digested with *Bln*I and *Nru*I to confirm successful cloning of the sgRNA and the correct sequence of the sgRNA (using primes P7/P8) confirmed by Sanger sequencing. An additional intermediate plasmid, pLf0130 (Supplementary Figure 1) was modified by replacing the existing promotor region of the *gapdh* gene (PF3D7_1462800) by the promoter region of *etramp10.3* promoter (PF3D7_1016900) of a reporter cassette containing mCherry fused to luciferase with the 3'UTR region of histidine-rich protein II (PF3D7_0831800; 626 bp obtained by digestion of an intermediate plasmid pLf0053 Mogollon et al., 2016 with restriction sites *Apa*I/*Xba*I). The *etramp10.3* promoter region (1.7kb) was amplified from *Pf* NF54 genomic DNA using the primers P9/P10 and cloned into pLf0130 using restriction sites *SacI*II/*Xho*I or *Kpn*I. Next, to obtain the complete *mcherry-luc@etramp10.3* expression cassette, pLf0130 was digested with *Apa*I/*Sac*II and this cassette was cloned in pLf0047 using the same enzymes, resulting in the final construct pLf0049.

To create the *mCherry-luc@gapdh* reporter line we used the Cas9 construct (pLf0019), described above, in combination with a sgRNA donor-DNA plasmid (pLf0048). The sgRNA-donor DNA construct (pLf0048) contains a h*dhfr-yfcu* drug-selectable marker (SM) cassette for selection with the drug WR99210. To generate pLf0048, an intermediate plasmid CM269 (Supplementary Figure 1) was modified by inserting the promoter region (1.6 kb) of the *gapdh* gene (PF3D7_1462800) that was PCR-amplified from *Pf* NF54 genomic DNA using the primers P11/P12 (Supplementary Table 1). CM269 was digested with restriction enzymes *Nru*I/*Xho*I and the *gapdh* promoter was cloned into CM269 using the same enzymes, resulting in construct pLf0130 (Supplementary Figure 1). Finally, to obtain the *mcherry-luc@gapdh* expression cassette pLf0130 was digested with *Apa*I/*Sac*II and this cassette was cloned in pLf0047 (see above) using the same enzymes, resulting in the final construct pLf0048 (Supplementary Figure 1).

Isolation of plasmids for transfection and transfection of synchronized ring stage parasites was performed as described (Mogollon et al., 2016) and parasites were transfected with a mixture of ~50 μg of each circular plasmid (Cas9 and sgRNA/Donor DNA construct). After transfection, parasite cultures were maintained under standard culture conditions in the semi-automated culture system (see above). Selection of transformed parasites was performed by applying “double” positive selection 24 h after transfection using the drugs WR99210 (2.6 nM) and BSD (5 μg/ml) as described (Mogollon et al., 2016). Drug pressure in the cultures was maintained until thin blood-smears were parasite-positive (usually after 14 to 26 days). Positive selection will select for the parasites that were transfected successfully with both plasmids (Cas9 and sgRNA/Donor constructs). Subsequently, both drugs were removed from the cultures for 2–4 days, followed by applying negative selection by addition of 5-Fluorocytosine as described (Mogollon et al., 2016) in order to eliminate parasites that retained the crRNA/Donor construct as episomal plasmid and enriching for the transfected parasites containing the donor DNA integrated into the genome. Negative drug pressure in the cultures was maintained until thin blood-smears were parasite-positive (usually after 7 days). After negative selection infected RBC (iRBC) were harvested from cultures with a parasitemia of 4 to 10% for genotyping by diagnostic PCR and Southern analysis (see next sections). Subsequently, selected parasites were cloned by limiting dilution as has been described previously (Mogollon et al., 2016). Cloned parasites were transferred in 10 ml culture flasks at 5% haematocrit and cultured under standard culture conditions (see above) in the semi-automated culture system for collection of parasites for further genotype and phenotype analyses (see next section).

### Genotyping of *mcherry-luc@etramp 10.3* and *mCherry-luc@gapdh* Parasites

For genotyping diagnostic PCR and Southern analysis of digested DNA were performed using DNA isolated from iRBC obtained from 10 ml cultures (parasitemia 3–10%). DNA was isolated as described (Mogollon et al., 2016). Correct integration of the donors constructs was analyzed by PCR amplification of the mCherry-luciferase gene (primers P13/P14), the fragment for 3' integration (3'int; primers P15/P16) and the *p47* gene (ORF; primers P17/P18) (see Supplementary Table 1 for primer sequences). The PCR fragments were amplified using Go-taq® DNA polymerase (Promega) following standard conditions with an annealing temperature of 56°C for 20 s and a elongation step of 72°C for 4 min. All other PCR settings were according to manufacturer's instructions.

Southern blot analysis of digested DNA was performed with *Hpa*I digested genomic DNA (4 h at 37°C). Digested DNA was hybridized with probes targeting the *p47* homology region 1 (HR1), amplified from *Pf* NF54 genomic DNA by PCR using the primers P1/P2 and a second probe targeting ampicillin (Amp) gene, obtained by digestion of the intermediate plasmid pLf0040 with *Aat*II/*Pvu*I (550bp).

### Phenotype Analysis of *mcherry-luc@etramp 10.3* and *mCherry-luc@gapdh* Parasites

#### Asexual Blood-Stages

The growth rate of asexual blood-stages (parasitemia) was monitored by determination of parasitemia in standard *in vitro* cultures for a period of 4 days with a starting parasitemia between 0.1 and 0.5%. Parasitemia was determined either by FACS analysis of iRBC or by counting parasitemia in Giemsa-stained thin blood films. For FACS analysis iRBC were stained with the DNA-specific dye Hoechst-33258 in 1 ml of PBS by adding 4 μl of a 500 μM stock-solution (final concentration 2 μM), as has been described previously (Mogollon et al., 2016). mCherry expression of asexual blood-stages was analyzed by standard fluorescence microscopy. In brief, 200 μl samples of iRBC were collected from 10 ml cultures with a parasitemia between 4 and 10% and stained with the DNA-specific dye Hoechst-33342 by adding 4 μl of a 500 μM stock-solution (final concentration 10 μM) for 20 min at 37°C. Subsequently, 5 μl was placed on a microscopic slide (mounted under a cover slip) and fluorescence of live iRBC analyzed using a Leica fluorescence MDR microscope (100x magnification). Pictures were recorded with a DC500 digital camera microscope using Leica LAS X software and with the following exposure times: mCherry 0.6 s; Hoechst-33342 0.136 s; bright field 0.62 s (1x gain). Luciferase expression in *mCherry-luc@etramp* asexual blood-stages was determined as follows. A serial dilution of asexual blood-stages was prepared from parasites samples that were collected (in triplicate) from asexual blood-stage cultures with a final number of 5 × 10^6^ parasites per sample. These were diluted with RPMI-1640 culture medium containing uninfected RBC (5% haematocrit) to prepare (triplicate) samples with 1 × 10^6^, 1 × 10^5^, 1 × 10^4^, 1 × 10^3^, 1 × 10^2^, 1 × 10^1^ parasites, respectively. Samples of 40 μl containing only uninfected RBC (5% haematocrit) were used as controls. The cells were pelleted by centrifugation (800 g; 30 s) and were lysed with 40 μl of cell culture Lysis 5X reagent from Promega (1 in 5 dilution in miliQ water). The complete lysates were collected in black 96-well plate (flat bottom) and luciferase activity was measured after adding 50 μl of the Luciferase substrate (Luciferase Assay System Promega). Luciferase activity (in relative light units; RLU) was measured using the Glomax multi detection system Luminometer (Promega) and the Instinct software (Promega).

#### Gametocytes

Gametocyte production (stage V male/female gametocytes) and exflagellation of male gametocytes were analyzed in gametocyte cultures established as described previously (van Dijk et al., 2010). For analysis of mCherry expression, stage II -V gametocytes were collected at days 7, 9, 11, and 14. Samples (200 μl) were collected, pelleted by centrifugation (800 g; 30 s) and stained with Hoechst-33342 and mCherry expression analysis using fluorescence microscopy as described for asexual blood-stages. Luciferase expression was determined in *mCherry-luc@etramp* gametocytes (stage III-IV) collected at day 11. A similar serial dilution of gametocytes was prepared as described for asexual blood-stages and luciferase activity in gametocytes was determined as described for the asexual blood-stages.

#### Oocysts and Sporozoites

For analysis of mosquito stages (oocysts and sporozoites), *Anopheles stephensi* mosquitoes were infected with day 14 gametocytes cultures using the standard membrane feeding assay (SMFA) (Ponnudurai et al., 1987, 1989). Oocysts numbers and mCherry expression in oocysts was determined at day 8 and 10 after infection. mCherry expression was analyzed using a Leica fluorescence MDR microscope (100x magnification). Pictures were recorded with a DC500 digital camera microscope using Leica LAS X software and with the following exposure times: mCherry 0.6 s; Hoechst-33342 0.136 s; bright field 0.62 s (1x gain). Collection of salivary gland sporozoites for counting numbers and expression of mCherry was performed at day 14 and 21 after feeding. For counting sporozoites, salivary glands from 30 to 60 mosquitoes were dissected, collected in 100 μl of RPMI-1640 pH 7.2 and homogenized using a grinder. Sporozoites were counted using a Bürker cell counter using phase-contrast microscopy. For mCherry expression, the isolated sporozoite were pelleted by centrifugation (800 g; 5 min). The pellet was suspended in 40 μl 1X PBS and sporozoites stained with Hoechst-33342 (10 μM) for 30 min at 37°C. Of this solution, 5 μl was placed on a microscopic slide (mounted under a cover slip) and fluorescence of sporozoites in live was analyzed using a Leica fluorescence MDR microscope (100x magnification). Pictures were recorded with a DC500 digital camera microscope using Leica LAS X software and with the following exposure times: mCherry 0.6 s; Hoechst-33342 0.136 s; bright field 0.62 s (1x gain).

Luciferase activity in *mCherry-luc@etramp* sporozoites was determined in duplicate samples (total volume of 40 μl of RPMI) of a serial dilution of 0.31 × 10^4^-5.0 × 10^4^ salivary glands sporozoites. Fifty microliter of D-Luciferin (0.4 mg/ml; Perkin Elmer Life Sciences, Waltham, USA) was added to the 40 μl of diluted sporozoites in a black 96-well plate (flat bottom). The *in vivo* imaging system Lumina (Caliper Life Sciences, USA) was used to measure luciferase activity. Imaging data were analyzed using the Living Image® 4.5.5 software (Caliper Life Sciences, USA). Bioluminescence images were acquired with a 12.5 cm field of view (FOV), medium binning factor and an “auto-exposure” time of maximum 2 min.

#### Liver-Stages

Liver-stages of *mCherry-luc@etramp* and *gfp-luc@eef1a* were cultured *in vitro* using cryopreserved primary human hepatocytes obtained from Tebu-bio (Tebu-bio.com–Life science Research) and thawed according to the instructions of Sekisui/Xenotech (Sekisui XenoTech, LLC; Kansas City). Cells were cultured in Williams's E culture medium supplemented with 10% FCS, 1% penicillin-streptomycin, 1% fungizone, 0.1 IU/ml insulin and 70 μM hydrocortisone 21-hemisuccinate (Sigma). Hepatocytes were seeded in Greiner clear bottom white 96-well plates at a density of 5 × 10^4^ cells per well, 2 days before infection with sporozoites as described previously (Boes et al., 2016). Sporozoites were collected from infected mosquitoes at day 21 after as described above and hepatocyte cultures (at 37°C) were infected with 5 × 10^4^ sporozoites per well. Three hours (h) after the addition of sporozoites, the cultures were washed three times with 1X PBS to remove mosquito material as well as sporozoites and complete Williams's E medium was added and cultures which were incubated overnight at 37°C. The day after, the culture medium was replaced and then was changed every 48 h until day 7.

Cultured cryopreserved primary human hepatocytes were infected with 5 × 10^4^ *mCherry-luc@etramp10.3* and *GFP-luc@eef1*α sporozoites per well of a 96-wells plate. Development of liver-stages of these two transgenic lines at day 2, 4, and 6 after infection was compared with WT liver-stages by immunofluorescence using antibodies against the cytoplasmic protein *Pf* HSP70 (rabbit, anti-*Pf* HSP70; 1:75 dilution of 1 mg/ml stock solution StressMarqBiosciences) and secondary antibody (goat anti-rabbit IgG AF594; 1:200 dilution of 4 mg/ml stock solution Invitrogen). Hepatocyte and parasite nuclei were stained with 300nM DAPI (Invitrogen D1306). Fluorescence signals were visualized using a Cytation Imager (Biotek, Winooski, VT). The percentage of infected hepatocytes, the size of the liver-stages and the intensity of *Pf* HSP70 staining were analyzed using the FIJI image analysis software package (Schindelin et al., 2012).

For analysis of mCherry expression in liver-stages, infected hepatocytes were fixed (at day 3, 4, and 5) with 4% paraformaldehyde in 1X PBS during 1 h at room temperature. After fixation the wells were washed three times with 1X PBS and permeabilized with 20 μl of 0.5% triton in 1X PBS and then blocked with 10% of FCS in 1X PBS for 1 h. Fixed cells were washed with 1X PBS and incubated with monoclonal antibodies against *PfHSP70* (rabbit, anti-*Pfhsp70*; 1:200 dilution of 100 μg/ml stock solution StressMarqBiosciences) and against mCherry (goat, anti-*mCherry* Mab Sicgen antibodies; 1:200 dilution of 3 mg/ml stock solution) for 1 h at room temperature. Subsequently, cells were rinsed 3 times with 1X PBS and incubated with the secondary antibodies Alexa Fluor®488/594-conjugated chicken anti-rabbit and anti-goat (Invitrogen Detection Technologies at 1:200). Finally, the cells were washed again three times with 1X PBS and stained with the DNA-specific dye Hoechst-33342 at a final concentration of 10μM. Fixed cells were covered with 1-2 drops of an anti-fading agent (Vectashield), and stained cells were analyzed for fluorescence using a Leica fluorescence MDR microscope (100x magnification). Pictures were recorded with a DC500 digital camera microscope using Leica LAS X software with the following exposure times: Alexa 488: 0.7s; Alexa 594: 0.6s; Hoechst 0.136s; bright field 0.62s (1x gain). Luciferase expression in liver-stages was monitored daily by adding 150 μl of Bright-Glo luciferase assay substrate (Promega, Madison, WI) to 150 μl of culture medium to each well and luminescence was quantified using a Synergy 2 multi-purpose plate reader (Biotek, Winooski, VT). Background was determined by measuring wells with uninfected hepatocytes.

The sensitivity of liver-stage development to atovaquone determined by measurement of luminescence of infected primary human hepatocytes maintained in 96-well plates and incubated with a serial dilution of atovaquone. Atovaquone was serially diluted in DMSO and then in Williams E culture medium to reach a final DMSO concentration of 0.1%. Atovaquone was added to the 96-well cultures 3hrs after adding 5x10^4^ sporozoites to cultures of primary human hepatocytes. Medium containing drug was refreshed each day and the cultures were allowed to proceed for 4 days after which luminescence was determined as describe above.

### Statistics

Data were analyzed using GraphPad Prism software package 5.04 (GraphPad Software, Inc). Significance difference analyses between *WT* and the reporter lines *mcherry-luc@etramp 10.3* and *gfp-luc@eef1a* was performed using the unpaired Student's *t*-test.

## Results and Discussion

**Figure 1:**
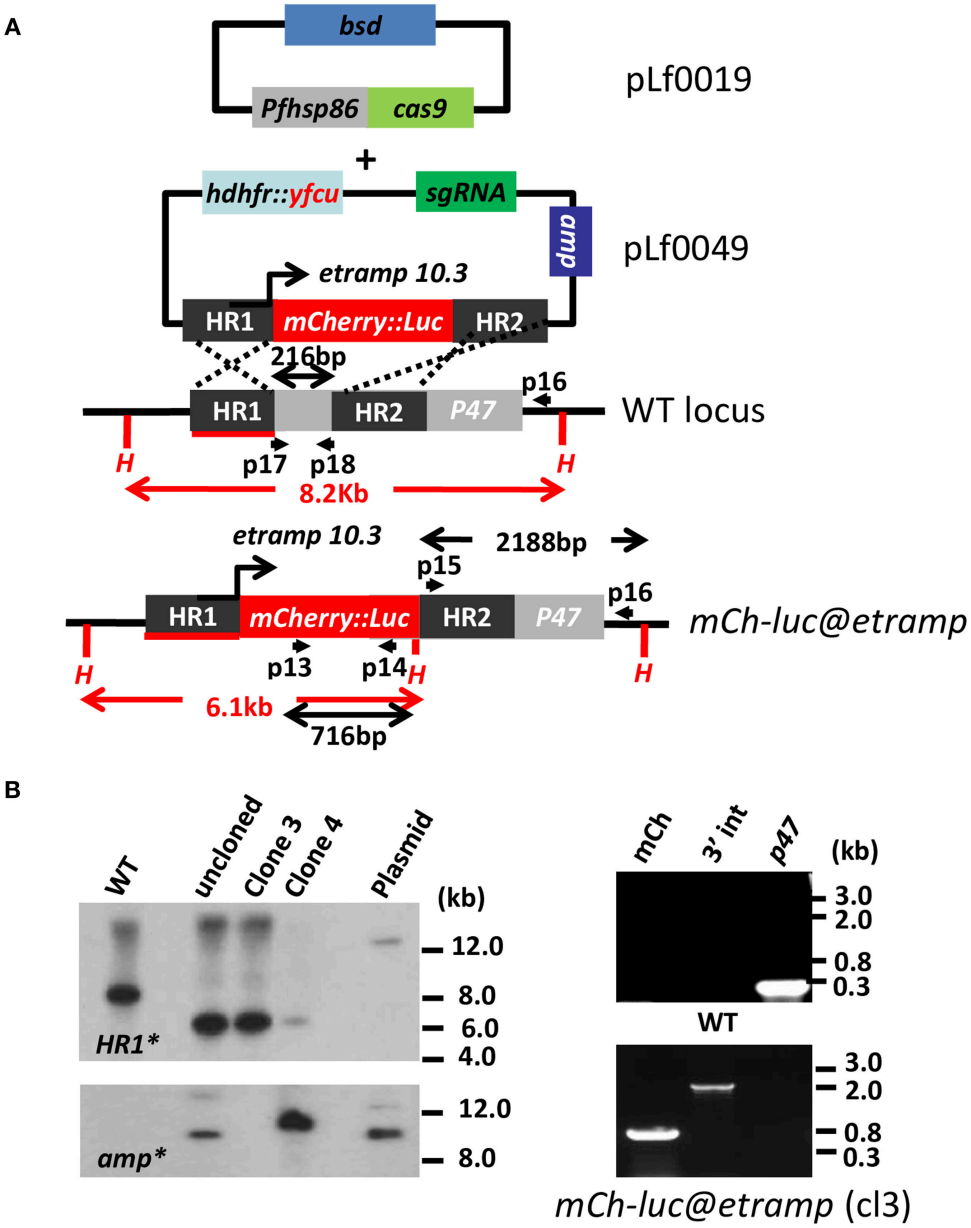
Generation and characterization of a *P. falciparum* reporter line expressing mCherry-luciferase under control of the *etramp10.3* promoter. **(A)** Schematic representation of the Cas9 (pLf0019) and sgRNA/donor (pL0049) constructs used to introduce the *mCherry-luciferase* expression cassette into the *P*. *falciparum p47* gene locus. The *mCherry-luciferase* fusion gene is under the control of the promoter of the *etramp10.3* gene. The *p47* homology regions (HR1, HR2) used to introduce the donor DNA (i.e., *the mCherry-luciferase* expression cassette), location of primers (p), sizes of restriction fragments (H: *Hpa*I; in red), and PCR amplicons (in black) are indicated. Primer sequences (shown in black and bold) are shown in Supplementary Table 1. WT–wild type; *bsd*–blasticidin selectable marker (SM); *hdhfr::yfcu*–SM in donor plasmid. *mCh-luc@etramp–*the final reporter line *mCherry-luc@etramp10.3*. **(B)** Southern analysis of *Hpa*I restricted DNA (left panel) and diagnostic PCR (right panel) to confirm correct integration of construct pLf0049 into the *p47* locus [wild type (WT), transfected, uncloned (uncl) parasites, clones 3 and 4 of *mCherry-luc@etramp* parasites, and plasmid (PL)]. Digested DNA was hybridized with a probe targeting the homology region 1 of *p47* [HR1; primers p3/p4; see **(A)**], showing the expected different-sized DNA fragments [shown in red in **(A)**] in WT (8.2 kb) and *mcherry-luc@etramp10.3* parasites (6.1 kb). The absence of hybridization of digested DNA with a probe for *ampicillin* (*amp*) confirms the absence of donor-DNA plasmid and single cross-over event integration of construct pLf0049 in clone 3. Diagnostic PCR of *mCherry-luc@etramp* clone 3 confirms the presence of the *mCherry* gene (lane 1; primers p13/p14; 716 bp), correct 3′ integration of the construct (lane 2; primers p15/p16; 2,188 bp) and absence of the *p47* gene (lane 3; primers p17/p18; 216 bp). Primer locations and product sizes are shown in **(A)** and primer sequences in Supplementary Table 1). Uncropped images of the PCR and Southern analyses are shown in Supplementary Figures 5, 6.

In order to create a *P. falciparum* parasite that has high reporter protein expression in sporozoites and/or liver-stages, we selected the promoter of the *etramp10.3* gene (PF3D7_1016900) to drive gene expression. We selected the *P. falciparum p47* gene (PF3D7_1346800) locus for introduction of the transgene expression cassette into the genome as it has been shown to be suitable for transgene expression without compromising parasite development in blood- and liver-stages as well as in *A. stephensi* mosquitoes (Talman et al., 2010; Vaughan et al., 2012; Vos et al., 2015). We had previously generated various transgenic reporter *P. falciparum* lines, where GFP-expression cassettes were introduced into the *p230p* gene locus (PF3D7_0208900), but disruption of this gene resulted in parasites that could not complete mosquito stage development (Marin-Mogollon et al., 2018). Using CRISPR/Cas9 methodology a transgenic *P. falciparum (Pf)* parasite line was created that encodes an *mCherry-luciferase* fusion gene under the control of 1.7 kb of 5' UTR of the *etramp10.3* gene. This expression cassette was introduced into the neutral *p47* gene locus (PF3D7_1346800) using a two plasmid CRISPR/Cas9 method; a previously described Cas9 construct (pLf0019) was used that contains the Cas9 expression cassette and a *blasticidin* (BSD) drug-selectable marker cassette (Mogollon et al., 2016) and a second plasmid (pLf0049) containing both the *p47* sgRNA and the donor DNA sequences (Supplementary Figure 1). The pLf0049 plasmid contains the *p47* targeting sequences, the *mCherry-luc@etramp10.3* expression cassette and a h*dhfr*-y*fcu* drug-selectable marker cassette (see Figure 1A and Materials and Methods section for details of the generation of the constructs). This plasmid is designed to introduce the reporter *mCherry-luc@etramp10.3* cassette into the *p47* gene locus by double cross-over homologous recombination to repair the double strand break (DSB) introduced into the *p47* gene locus (Figure 1A).

As a control line we created also a transgenic *P. falciparum (Pf)* parasite line that contains an *mCherry-luciferase* fusion gene under control of 1.6kb 5'UTR of the gene encoding the glycolytic enzyme, *gapdh* (PF3D7_1462800). Recently we have shown that this promoter drives high GFP expression in *P. falciparum* blood-stages (Mogollon et al., 2016). The same Cas9 construct, pLf0019, was used in combination with a plasmid (pLf0048) expressing the *p47* sgRNA and *p47* donor-DNA sequences (Supplementary Figure 1). This plasmid is designed to introduce the reporter *mCherry-luc@gapdh* and the h*dhfr*-y*fcu* drug-selectable marker cassettes into the *p47* gene locus by double cross-over homologous recombination to repair the double strand break (DSB) introduced into the *p47* gene locus (see Supplementary Figure 2A and Materials and Methods section for details of the generation of the pLf0048 construct).

Transfection of *Pf* wild type (WT) NF54 parasites was performed using synchronized ring-stage parasites that were transfected with ~50 μg of each circular plasmid (Cas9 and sgRNA/donor-DNA constructs; see Materials and Methods section). Selection of transfected parasites containing both plasmids was performed by applying “double” positive selection with the drugs BSD and WR99210 until parasites were detectable by thin blood-smear analysis (between day 14 to 26 post transfection). Subsequently, parasites were cultured for 2–4 days without drugs, followed by the application of negative (5-FC) selection to eliminate parasites that retain transfection constructs (i.e., donor-DNA) as episomal plasmids and to enrich for parasites in which the donor-DNA construct has integrated into the parasite genome. Subsequently, drug-selected parasites were cloned by limiting dilution. Genotyping of two clones of the *mCherry-luc@etramp10.3* line (exp.0064 cl3 and cl4) and four clones of the *mCherry-luc@gapdh* line (exp.0055cl2, 3, 4 and 6) by Southern analysis and PCR revealed integration of the *mCherry-luciferase* cassettes into the *p47* locus (Figure 1B; Supplementary Figure 2B). Whereas, in all clones of *mCherry-luc@gapdh* the donor DNA appears to be integrated by double cross-over integration, plasmid DNA is present in clone 4 of *mCherry-luc@etramp10.3* line (Figure 1B) indicating single cross-over integration of pLf0049 in this clone. For further phenotype characterization of the cloned transgenic lines we selected mCherry-luc@etramp10.3 clone 3 and *mCherry-luc@gapdh* clone 2. PCR analyses of these two clones confirmed correct integration of the expression cassettes into the p47 gene locus (Figure 1B; Supplementary Figure 2B). *In vitro* growth of asexual blood-stages of clones of both lines was comparable to the growth of asexual blood-stages of the parent wild type (WT) NF54 strain (Supplementary Figure 2C) and the two clones of both lines produced WT-comparable numbers of mature stage V male and female gametocytes in standardized gametocyte cultures (Supplementary Table 2).

### Examination of mCherry and Luciferase Expression of *mCherry-luc@etramp10.3* and *mCherry-luc@gapdh* in Blood-Stages

**Figure 2:**
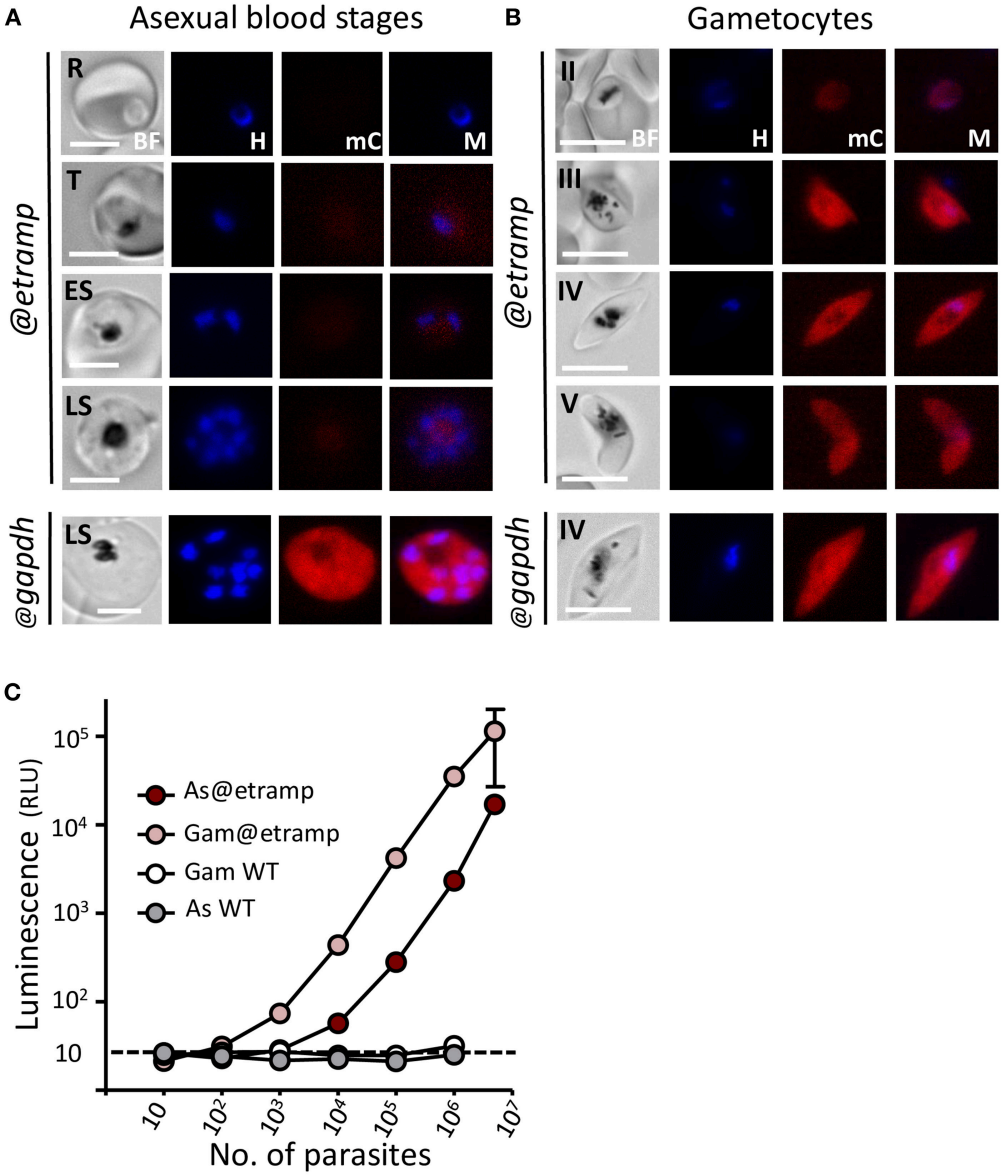
Expression of mCherry and luciferase in asexual blood-stages and gametocytes of *mcherry-luc@etramp10.3* and *mcherry-luc@gapdh* parasites. **(A)** Fluorescence microscopy analysis of live *mcherry-luc@etramp10.3* asexual blood-stages (@*etramp*). No mCherry fluorescence signal above background were detected in the different stages. R, rings; T, trophozoites; ES, early schizonts; LS, late schizonts. Lower panel, an mCherry-positive schizont of the control line *mcherry-luc@gapdh* (@*gapdh*). See Supplementary Figure 3A for other asexual stages of this control line. Nuclei were stained with Hoechst-33342. All pictures were recorded with standardized exposure/gain times to visualize differences in fluorescence intensity [mCherry 0.7 s; Hoechst 0.136 s; bright field 0.62 s (1x gain)]. Bright field (BF), Hoechst (H), mCherry (mC), Merge (M). Scale bar, 4 μm. **(B)** Fluorescence microscopy analysis of mCherry expression in live *mcherry-luc@etramp10.3* gametocytes (@*etramp*). Gametocyte stage II, III, IV and V are shown. Lower panel, stage IV gametocyte of the control line mcherry-luc@gapdh (@*gapdh*). See Supplementary Figure 3B for other gametocyte stages of this control line. Nuclei were stained with Hoechst-33342. All pictures were recorded with standardized exposure/gain times to visualize differences in fluorescence intensity [mCherry 0.7 s; Hoechst 0.136 s; bright field 0.62 s (1x gain)]. Bright field (BF), Hoechst (H), mCherry (mC), Merge (M). Scale bar, 7 μm. **(C)** Correlation between luciferase expression (luminescence, RLU, photons/sec) and number of parasites in serial dilutions series of asexual blood-stages (AS) and gametocytes stage III/IV (Gam) of the *mcherry-luc@etramp10.3* line (3 independent experiments). Wild type *Pf* NF54 parasites (WT) were used as a control. Black dotted line: luminescence value of uninfected cells. The mean luminescence value of triplicate samples is shown; error bars represent the standard deviation. Correlation coefficient r (two-tailed Spearman's test: 10^3^-10^7^ parasites): 1.00; *p* = 0.016 for AS and 1.0; *p* = 0.016 for Gam.

We analyzed mCherry expression in the reporter parasites by fluorescence-microscopy of cultured asexual blood-stages. The *mCherry-luc@etramp10.3* line did not exhibit clear mCherry expression in asexual blood-stages (ring-forms, trophozoites, and schizonts) and the fluorescent signals were indistinguishable from background of uninfected red blood cells (Figure 2A). In contrast, asexual blood-stages of the control *mCherry-luc@gapdh* line showed mCherry expression, in agreement with our previous analyses of GFP expression in blood-stages of the GFP@*gapdh* line, where the gene encoding GFP was under the control of the *gapdh* promoter (Mogollon et al., 2016) (Figure 2A; Supplementary Figure 3A).

Gametocytes of *mCherry-luc@gapdh* were mCherry positive, again in agreement with our previous observations of the GFP@*gapdh* parasites (Marin-Mogollon et al., 2018) (Figure 2B; Supplementary Figure 3B). However, mCherry signals in gametocytes were relatively weak in all stages during their development (stage II-IV). The *mCherry-luc@etramp10.3* parasites exhibit strong mCherry expression in stage III-V gametocytes (Figure 2B), with weak mCherry signals being detectable in at least 20% of stage II gametocytes, increasing to more than 95% of stage III-V gametocytes that were strongly mCherry positive. The high *etramp10.3* promoter activity in gametocytes is in agreement with the high levels of *etramp10.3* transcripts and ETRAMP10.3 protein previously reported in gametocytes in genome-wide transcriptomic and mass spectrometry analyses. Peak *etramp10.3* transcript abundance was observed in stage III gametocytes (López-Barragán et al., 2011) and ETRAMP10.3 peptides were detected in proteomic analyses of mature male and female gametocytes (Lasonder et al., 2016; Miao et al., 2017), with higher protein expression in gametocytes than in asexual blood-stages.

The absence of clear mCherry signals in *mCherry-luc@etramp10.3* asexual blood-stages was unexpected since expression of ETRAMP10.3 in asexual blood-stages had been detected by mass spectrometry-based proteomics (Florens et al., 2002; Silvestrini et al., 2010) and also been confirmed by immunofluorescence analysis using anti-ETRAMP10.3 antibodies (Mackellar et al., 2010). Moreover, unsuccessful attempts to delete the *P. falciparum etramp10.3* gene indicates that it is essential during asexual blood-stage development (Mackellar et al., 2010). It is possible that fluorescence microscopy analysis is too insensitive to detect low level mCherry expression from the mCherry-luciferase fusion protein in asexual blood-stages. We therefore performed luminescence assays to analyse luciferase expression in *mCherry-luc@etramp10.3* asexual blood-stages. Luminescence signals in parasites obtained from pure asexual blood-stage cultures were significantly higher than those of uninfected red blood cells (*p* < 0.0005 in culture wells with more than 10^4^ parasites; Figure 2C), indicating that asexual blood-stages express the mCherry-luciferase protein. Stage IV/V gametocytes obtained from gametocyte cultures had on average 30-fold higher luminescence values (3 exp.; range 15–60 fold) compared to asexual blood-stages. The luminescence values obtained from *mCherry-luc@etramp10.3* gametocyte (IV/V) or mixed asexual stage dilution series demonstrate a linear relationship between the number of parasites and signal intensity in the range of 1 × 10^3^ to 1 × 10^7^ parasites for gametocytes and 1 × 10^4^ to 1 × 10^7^ for asexual blood-stage parasites (Figure 2C). Combined our observations show that *mCherry-luc@etramp10.3* parasites have high level expression of mCherry and luciferase in stage III-V gametocytes and low-level expression of luciferase in asexual blood-stages.

The essential role of ETRAMP10.3 during asexual blood-stage development and its expression in asexual blood-stages and gametocytes is in contrast to expression of the UIS4 protein of the rodent parasites, *P. berghei* and *P. yoelii*. The *P. berghei* and *P. yoelii uis4* gene is dispensable for blood-stage development s (Kaiser et al., 2004; Mueller et al., 2005) and there is no evidence that *uis4* is expressed in blood-stages either from proteomic or transcriptomic analyses [www.PlasmoDB.org; (Otto et al., 2014)]. In studies using transgenic parasites expressing fluorescent and/or luminescent proteins either under control of the *uis4* promoter or fused to UIS4 we are unable able to detect these reporters in blood-stage parasites (Grützke et al., 2014; Hopp et al., 2015). In contrast UIS4 has been show to play a vital role during liver-stage development in both *P. berghei and P. yoelii* (Kaiser et al., 2004; Mueller et al., 2005). In a previous study, where the *P. yoelii uii4* gene was replaced with the *etramp10.3* gene, it was demonstrated that ETRAMP10.3 is unable to complement the function of UIS4 in *P. yoelii* liver-stages (Mackellar et al., 2010). Combined, these observations indicate that UIS4 and ETRAMP10.4 may have different, or only partially overlapping, roles in rodent and human malaria parasites.

### Examination of mCherry and Luciferase Expression of *mCherry-luc@etramp10.3, mCherry-luc@gapdh*, and *GFP-luc@eef1α* in Oocysts and Sporozoites

**Figure 3:**
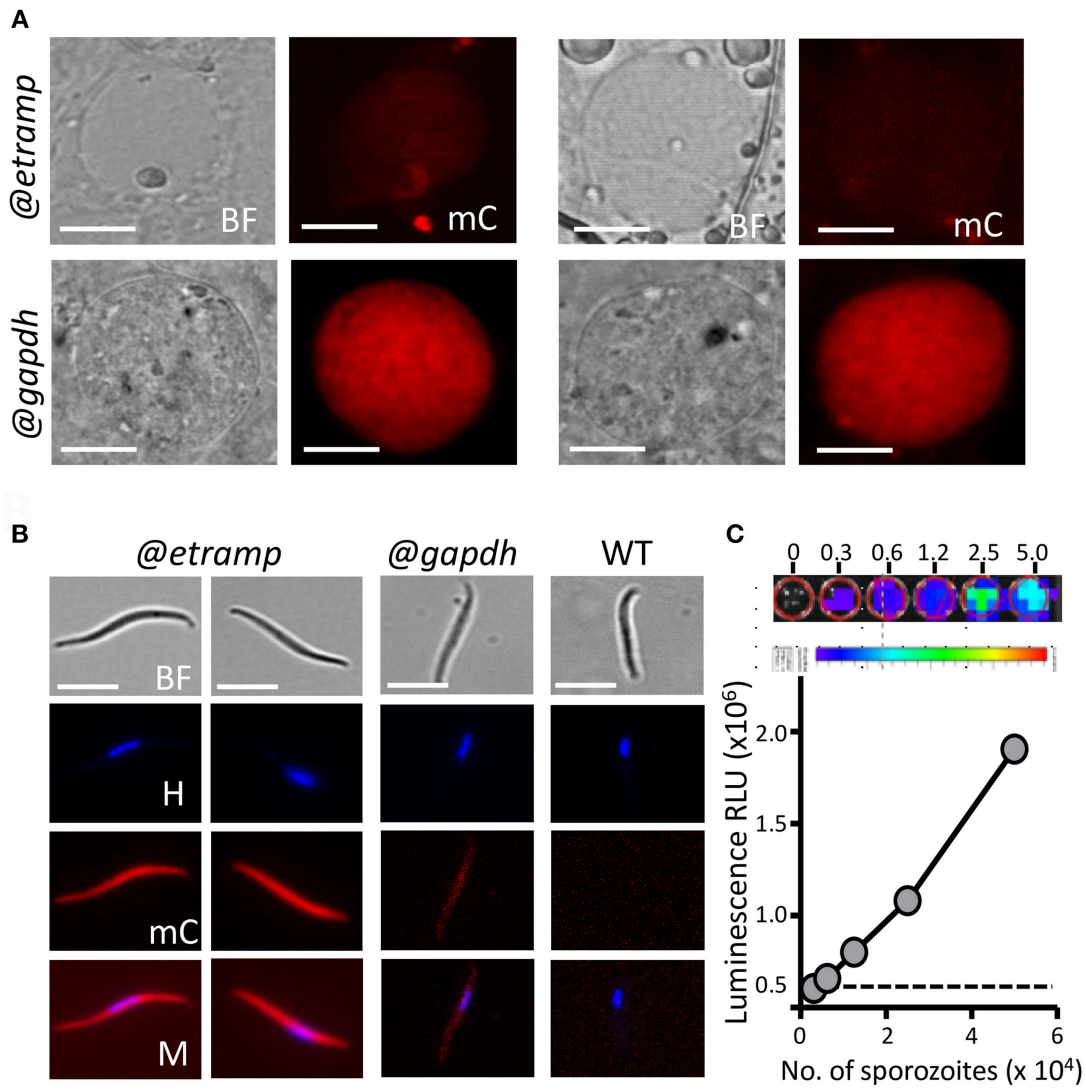
Expression of mCherry and luciferase in oocyst and sporozoites of *mcherry-luc@etramp10.3* and *mcherry-luc@gapdh* parasites. **(A)** Upper panel: Representative mCherry-fluorescence microscopy image of a live *mcherry-luc@etramp10.3* oocyst in *A. stephensi* mosquitoes at day 10 after infection (@*etramp*). No mCherry fluorescence signal above background was detected in oocysts. Lower panel: Representative mCherry-fluorescence microscopy image of a live oocysts of the control line *mcherry-luc@gapdh* (@*gapdh*). Bright field (BF), mCherry (mC). Scale bar, 20 μm. **(B)** Representative mCherry-fluorescence microscopy images of live salivary glands sporozoites collected at day 21 after infection of mosquitoes. Left panel: *mcherry-luc@etramp10.3* (@*etramp*) sporozoites. Middle panel: sporozoites of the control line *mcherry-luc@gapdh* (@*gapdh*). Right panel: wild type (WT) *Pf* NF54 sporozoites. Nuclei were stained with Hoechst33342. Bright field (BF), Hoechst (H), mCherry (mC), Merge (M). Scale bar, 7 μm. **(C)** Correlation between luciferase expression (luminescence, RLU) and number of sporozoites in serial dilutions series (in duplicate) of live *mCherry@etramp10.3* salivary gland sporozoites. Upper panel: Representative luminescence measurements of live sporozoites determined in 96-well plates. From left to right, rainbow images of a serial dilution of sporozoites (from 0, 0.3, 0.6, 1.2, 2.5, and 5.0 × 10^4^ sporozoites). Relative levels of luminescence range from low (blue), to medium (green), to high (yellow/red). Lower panel: Correlation between luciferase expression (luminescence, RLU, photons/sec) and number of sporozoites. Black dotted line: luminescence values of wells without sporozoites. The mean luminescence values of duplicate samples is shown; error bars represent the standard deviation. Correlation coefficient r (two-tailed Spearman's test): 0.99; *p* = 0.016.

We examined mCherry expression in oocysts and sporozoites, collected from *Anopheles stephensi* mosquitoes, which were fed with *mCherry-luc@etramp10.3* gametocytes using the standard membrane feeding assay. This line produced oocysts and sporozoites in the same range as wild type (WT) NF54 parasites (Supplementary Table 2). Weak mCherry signals were detected in maturing *mCherry-luc@etramp10.3* oocysts containing sporozoites (at day 8 and 11 after feeding; Figure 3A), but mCherry signals were not above the background mCherry signals observed with WT oocysts. In contrast, the oocysts (day 8) of the control line *mCherry-luc@gapdh* were clearly mCherry-positive (Figure 3A).

**Figure 4:**
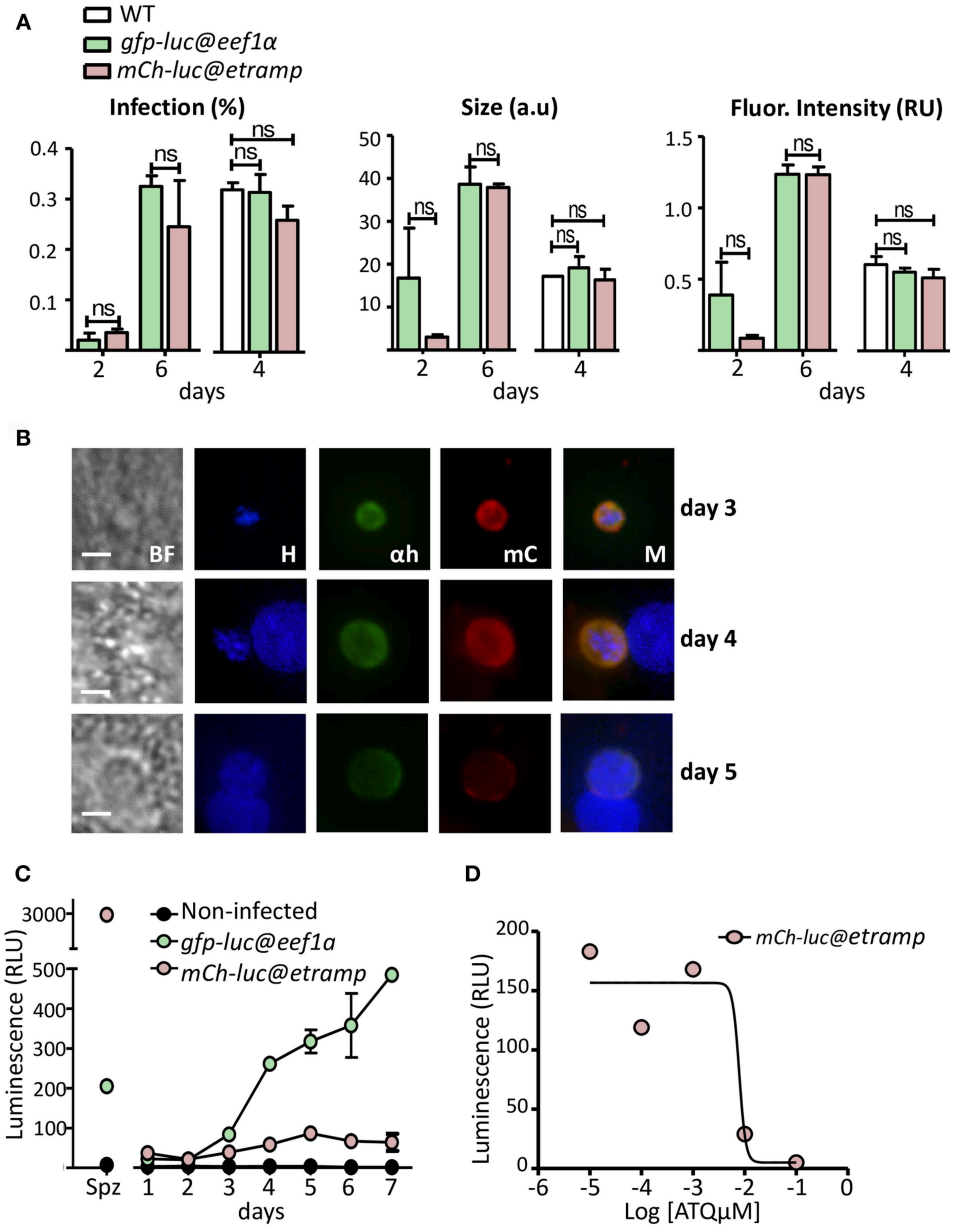
Liver stage development of *mcherry-luc@etramp10.3* parasites and expression of mCherry and luciferase. **(A)** Development of liver-stages of *mCherry-luc@etramp10.3* in cultured cryopreserved primary human hepatocytes and comparison with wild type (WT) and *GFP-luc@eef1*α. Shown are the percentage of infected hepatocytes (left graph), the size of liver-stages (middle graph; mean surface area; arbitrary units - a.u.) and the fluorescence intensity of *Pf* HSP70 staining (right graph; arbitrary units a.u. × 10^6^). Hepatocytes were infected with 5 × 10^4^ sporozoites and liver-stage development was analyzed at day 2, 4, and 6 after infection. At least 20 parasites were assessed at each time point. nd: not determined. Significance values (unpaired two-tailed *t*-test): n.s.–not significant. **(B)** Representative mCherry-fluorescence microscopy images of a fixed *mcherry-luc@etramp10.3* liver stages at day 3, 4, and 5 after infecting cryopreserved primary human hepatocytes with sporozoites. Fixed hepatocytes were stained with rabbit anti-*Pf* HSP70 and goat anti-mCherry antibodies. Secondary conjugated antibodies used: anti-IgG from rabbit Alexa Fluor® 488 (green) or anti-IgG from goat Alexa Fluor® 594 (red). Nuclei stained with Hoechst-33342. All pictures were recorded with standardized exposure/gain times; Alexa Fluor® 488 (green) 0.7 s; anti-IgG Alexa Fluor® 594 (red) 0.6 s; Hoechst (blue) 0.136 s; bright field 0.62 s (1x gain). Bright field (BF), Hoechst (H), anti-*Pf* HSP70 (αh), mCherry (mC), Merge (M). Scale bar, 10 μm. mCherry fluorescence was analyzed using *mcherry-luc@etramp10.3 cl3* in two independent experiments. **(C)** luciferase expression (luminescence, RLU, photons/sec) in sporozoites (spz) and in liver-stages from *mCherry-luc@etramp10.3 cl3* and the control line *gfp-luc@eef1a*. Cultured cryopreserved primary human hepatocytes were infected with 5 × 10^4^ sporozoites and luminescence was measured during a 7 days period. Uninfected hepatocytes were used as a control (Black circle). The mean luminescence value of duplicate samples is shown; error bars represent the standard deviation. **(D)** Sensitivity of *mCherry-luc@etramp10.3* liver-stages to atovaquone ([ATQμM], concentration in micromolar). Inhibition of liver-stage development was determined in a single experiment by measurement of luminescence at day 4 after infection of cultured cryopreserved primary human hepatocytes with 5 × 10^4^ sporozoites. The IC_50_ value (7.9 nM) was calculated by non-linear regression using GraphPad Prism software package 5.04.

In contrast to the mCherry negative *mCherry-luc@etramp10.3* oocysts, *mCherry-luc@etramp10.3* sporozoites collected from salivary glands exhibited strong mCherry expression, whereas lower mCherry signals were detected in *mCherry-luc@gapdh* sporozoites (Figure 3B; Supplementary Figure 4). The activity of the *etramp10.3* promoter in salivary gland sporozoites is in agreement with detection of multiple ETRAMP10.3 peptides in *P. falciparum* sporozoite proteomes (Lindner et al., 2013; Swearingen et al., 2016). Expression of luciferase in *mCherry-luc@etramp10.3* sporozoites was examined and luminescence signals from a dilution series of purified sporozoites exhibit a linear relationship between sporozoite number and luminescence intensity in the range of 1.25 × 10^4^ to 5 × 10^4^ sporozoites (Figure 3C). We compared the luminescence signals of *mCherry-luc@etramp10.3* sporozoites (5 × 10^4^) with that those of a previously described transgenic sporozoite line that expresses GFP-luciferase fusion protein under control of the *eef1*α promoter (*GFP-luc@eef1*α) (Vaughan et al., 2012). The luminescence signal of *mCherry-luc@etramp10.3* sporozoites was 14-fold higher than the luminescence signal obtained from the same number of *GFP-luc@eef1*α sporozoites (Figure 4).

### Examination of mCherry and Luciferase Expression of *mCherry-luc@etramp10.3* and *GFP-luc@eef1α* in Liver-Stages

We next analyzed reporter expression in liver-stages; however, since *mCherry-luc@gapdh* sporozoites showed low mCherry expression, we compared *mCherry-luc@etramp10.3* liver-stages directly with *GFP-luc@eef1*α parasites as it has been reported that sporozoites and liver-stages of this line expresses both GFP and luciferase and this line has been used to analyse *Pf* liver stage development (Vaughan et al., 2012; Sack et al., 2014; Flannery et al., 2018; Foquet et al., 2018). Cultured cryopreserved primary human hepatocytes were infected with 5x10^4^ *mCherry-luc@etramp10.3* or *GFP-luc@eef1*α sporozoites per well of a 96-wells plate. Development of liver-stages at day 2, 4, and 6 after infection of the two transgenic lines and WT liver-stages was examined by immunofluorescence using a rabbit polyclonal antibody against the cytoplasmic *P. falciparum* protein PfHSP70. The percentage of infected hepatocytes, the size of the liver-stages and the intensity of HSP70 staining were comparable between the transgenic and WT parasites at day 4 after infection and liver-stages of both transgenic lines were comparable at day 6 (Figure 4A).

Expression of mCherry in *mCherry-luc@etramp10.3* liver-stages was analyzed at day 3, 4, and 5 after hepatocyte infection by immunofluorescence assay using anti-mCherry antibodies; mCherry signals were detectable in every Hsp70 positive-liver stage parasite on all days (Figure 4B). Expression of luciferase in liver-stages was also examined by luminescence assays (Figure 4C; in duplicate) and we compared the luminescence signals of *mCherry-luc@etramp10.3* with *GFP-luc@eef1*α at the same point of development. The luminesce signals of *mCherry-luc@etramp10.3* liver-stages, while significantly higher than background levels with a peak of expression at day 5 (unpaired *T*-test:^***^p ≤ 0.0001), were between 2.2 to 7.6 fold lower than *GFP-luc@eef1*α from day 3 to 7 (Figure 4C).

To determine if *mCherry-luc@etramp10.3* parasites could be used in a plate-based assay to test drug sensitivity of liver-stage parasites we performed a drug assay using atovaquone, which has potent activity against intra-hepatic parasites (Baragaña et al., 2015). The *in vitro* inhibition of *P. falciparum* liver-stage development was determined by measuring luminescence signals of *mCherry-luc@etramp10.3*-infected hepatocytes maintained in 96-well plates and incubated with different concentrations of atovaquone (0.001–100 nM). Atovaquone was added to the 96-well cultures of primary human hepatocytes 3 h after the addition of 5 × 10^4^ sporozoites and the cultures were allowed to proceed for 4 days, after which luminescence was determined. In this assay we determined an IC_50_ value of atovaquone of 7.9 nM (Figure 4D), which is comparable to the previously established IC_50_ value of atovaquone against liver-stages (Delves et al., 2012).

Combined, our observation show high mCherry and luciferase expression in gametocytes and sporozoites of the *mCherry-luc@etramp10.3* line. Although both mCherry and luciferase expression is detected in liver stages, luciferase expression driven by the *etramp10.3* promoter is lower than luciferase expression under the *eef1*α promoter.

In summary: As with rodent malaria parasites the development and expansion of reporter lines in *P. falciparum* would increase the range of analyses that can more easily be performed, both to interrogate *P. falciparum* gene function at different points of development and to permit the miniaturization and rapid screening of compounds or immune sera that target the parasite at different points of development. Specifically, the expression of mCherry (red) instead of GFP (green) can be an critical advantage, especially when transgenic parasites/sporozoites will be used in assays using GFP-expressing host cells. In addition, parasites expressing mCherry in gametocytes will be valuable tools in cross-fertilization assays between GFP-expressing and mCherry expressing parasites (Ukegbu et al., 2015, 2017) for gaining a better understanding of parasite fertilization and mosquito-stage development.

The knowledge of expression of reporters under different promoters, such as the *etramp10.3*, can be useful for generation of additional (gene knock-out/mutated) mutants that require the expression of transgenes in specific life cycle stages. In contrast to the high expression in gametocytes and sporozoites our studies indicate that the *etramp10.3* promoter is less suitable for high reporter expression in liver stages, which is unexpected based on the use of the *uis4* promoter of rodent parasites. We demonstrate that the reporter expression in sporozoites under control of the *etramp10.3* promoter is much higher than under control of the *eef1a* promoter and therefore this line can be a valuable tool for studies on the biology of *P. falciparum* sporozoites, for example to visualize sporozoite migration studies in human skin explants (performed in our laboratory) or to visualize and quantify *P. falciparum* sporozoite invasion of human hepatocytes.

## Author Contributions

CM-M, CJ, and SK came up with the study concept and design. CM-M, AS, KK, JB, FP, SM, TI, AO, JR, G-JG, SC-M, and BF-F acquired the data. CM-M, CJ, SK, KD, and BF-F conducted analysis and interpretation of the data. CM-M, CJ, and SK wrote the draft of the manuscript. CM-M, AS, BF-F, KD, JB, CJ, SK, RS, and AH critically revised the manuscript for important intellectual content. CM-M, AS, FP, JR, AO, G-JG, HK, SC-M, SM, and BF-F provided technical and/or material support. CJ and SK supervised the study. All authors reviewed the manuscript.

### Conflict of Interest Statement

KD, RS, KK, and JB are employed by the company TropIQ Health Sciences. The remaining authors declare that the research was conducted in the absence of any commercial or financial relationships that could be construed as a potential conflict of interest. The handling editor declared a past co-authorship with one of the authors SK.
