# Supplementary material for: A P. falciparum NF54 Reporter Line Expressing mCherry-Luciferase in Gametocytes, Sporozoites, and Liver-Stages
Source: Front Cell Infect Microbiol. 2019 Apr 16;9:96. doi: 10.3389/fcimb.2019.00096 (PMC6477837; doi:10.3389/fcimb.2019.00096)
Supplement: Supplementary file 1 [file Data_Sheet_1.PDF]

Supplementary Material

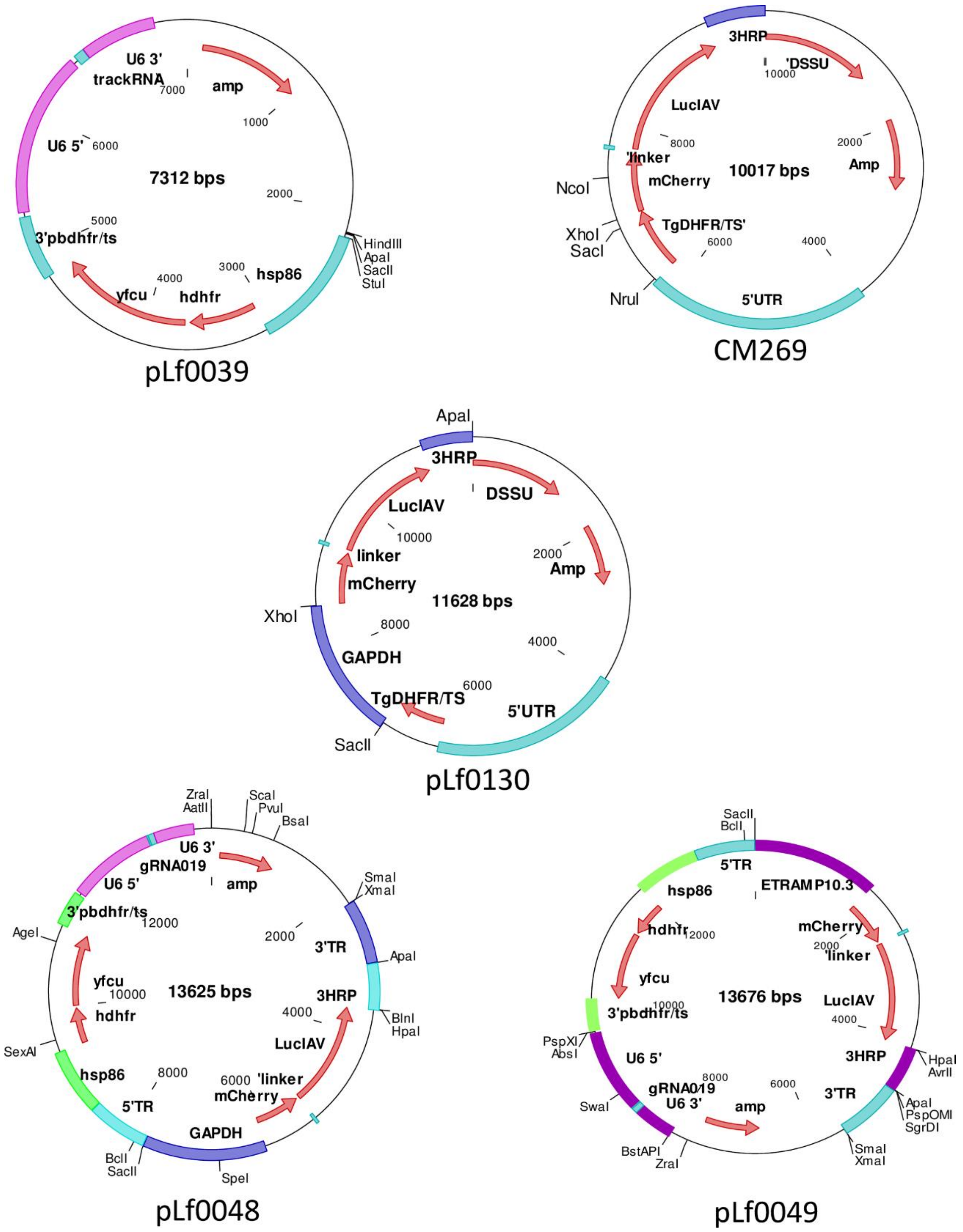

Supplementary Figure 1: Vector maps of DNA plasmids used to generate the *mCherry-luc@etramp10.3* and *mcherry-luc@gapdh* parasite lines

Vector maps of the different plasmids used to generate the *P. falciparum mCherry-luc@etramp10.3* and mCherry-luc@*gapdh* lines. See **Materials and Methods section** for description and details of the generation of these plasmids.

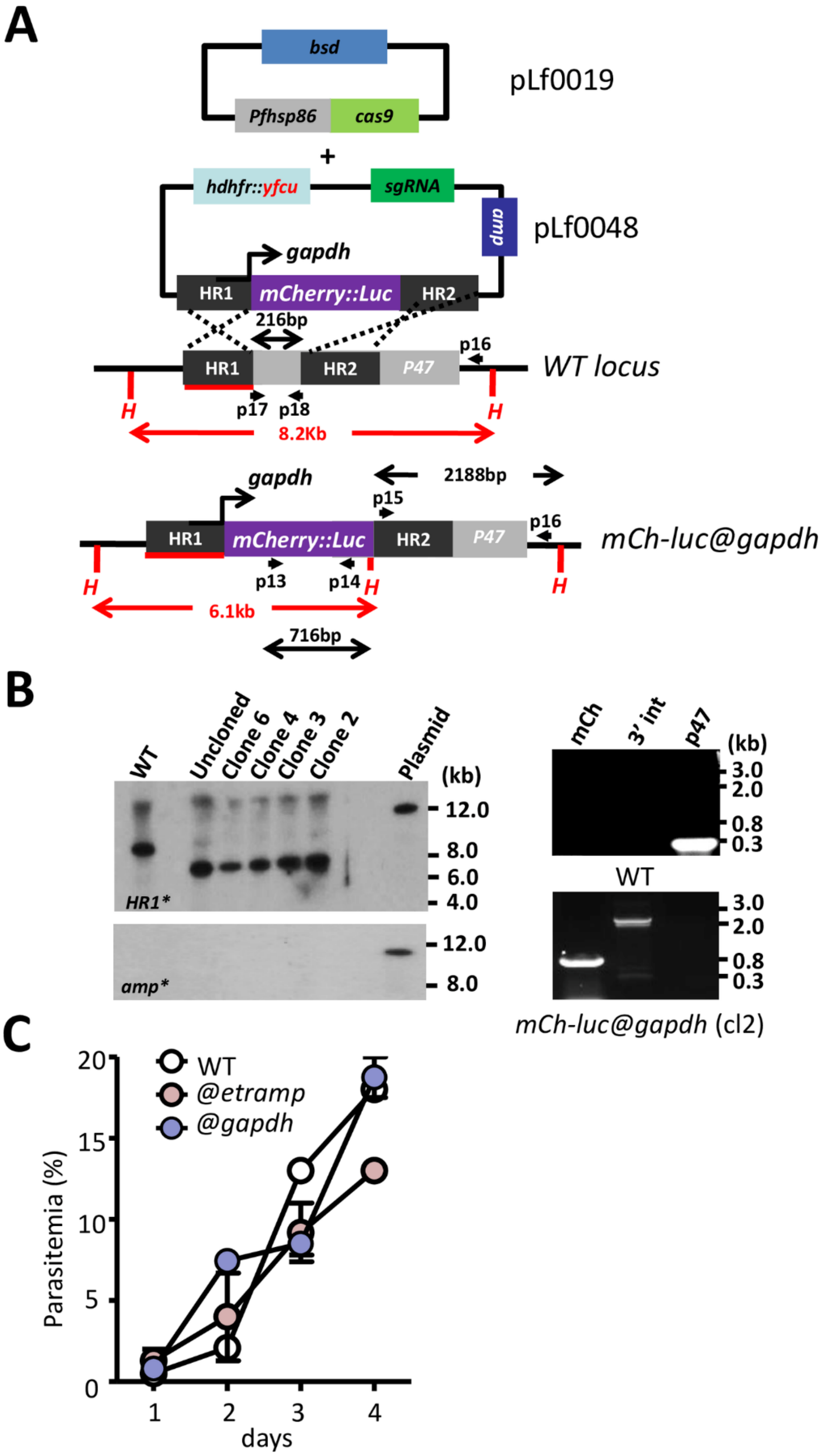

Supplementary Figure 2. Generation and characterisation of a *P. falciparum* reporter line expressing mCherry-luciferase under control of the *gapdh* promoter

(A) Schematic representation of the Cas9 (pLf0019) and sgRNA/donor (pL0048) constructs used to introduce the *mCherry-luciferase* expression cassette into the *P. falciparum p47* gene

locus. The *mCherry-luciferase* fusion gene is under the control of the promoter of the *gapdh* gene. *p47* homology regions (HR1, HR2) used to introduce the donor DNA (i.e. *the mCherry-luciferase* expression cassette), location of primers (p), sizes of restriction fragments (H: *Hpa*I; in red) and PCR amplicons (in black) are indicated. Primer sequences (shown in black and bold) are shown in **Supplementary Table 1**. WT – wild type; *bsd* – blasticydin selectable marker (SM); *hdhfr::yfcu* – SM in donor plasmid. *mCh-luc@gapdh* – the final reporter line *mCherry-luc@gapdh*.

(B) Southern analysis of *Hpa*I restricted DNA (left panel) and diagnostic PCR (right panel) to confirm correct integration of construct pLf0048 into the *p47* locus (wild type (WT), transfected, uncloned (uncl) parasites, clones 2, 3, 4 and 6 of *mCh-luc@gapdh* parasites and plasmid (PL)). Digested DNA was hybridized with a probe targeting the homology region 1 of *p47* (HR1; primers p3/p4; see (**A)**) showing the expected different-sized DNA fragments (shown in red in (**A)**) in WT (8.2kb) and *mcherry-luc@gapdh* parasites (6.1 kp). The absence of hybridisation of digested DNA with a probe for *ampicillin* (*amp*) confirms absence of donor-DNA plasmid and single cross-over events. Diagnostic PCR of *mCh-luc@gapdh* clone 2 confirms the presence of the *mCherry* gene (lane 1; primers p13/p14; 716 bp), correct 3' integration of the construct (lane 2; primers p15/p16; 2188 bp) and absence of the *p47* gene (lane 3; primers p17/p18; 216 bp). Primer locations and product sizes are shown in (**A)** and primer sequences in **Supplementary Table 1**). Uncropped images of the PCRs and Southern are shown in **Supplementary Figures 5 and 6**.

(C) Growth of asexual blood-stages of the *mcherry-luc@etramp10.3* line (clone 3; *@etramp*), the *mcherry-luc@gapdh* line (clone 4; *@gapdh*) and WT parasites in semi-automated culture system for a period of 4 days. Cultures were initiated with a parasitemia of 0.5%.

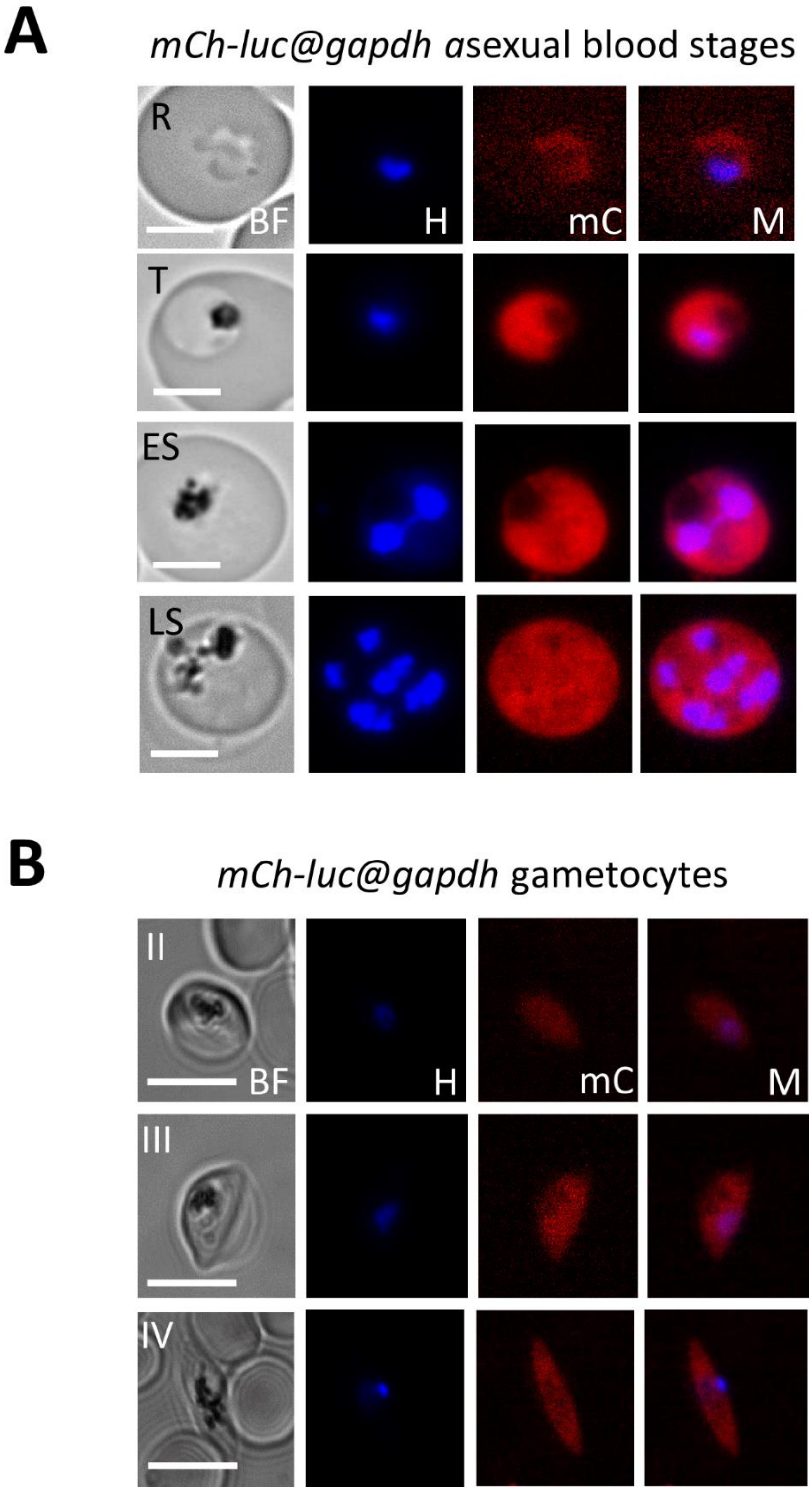

Supplementary Figure 3. Expression of mCherry in asexual blood-stages and gametocytes of *mcherry-luc@gapdh* parasites

(A) Fluorescence microscopy analysis of live *mcherry-luc@gapdh* asexual blood-stages. mCherry fluorescence signal above background were detected in the different stages. R: rings; T: trophozoites; ES: early schizonts; LS: late schizonts. Nuclei were stained with Hoechst-33342. All pictures were recorded with standardized exposure/gain times to visualize differences in fluorescence intensity (mCherry 0.7 s; Hoechst 0.136 s; bright field 0.62 s (1x gain)). Bright field (BF), Hoechst (H), mCherry (mC), Merge (M). Scale bar, 4µm.

(B) Fluorescence microscopy analysis of mCherry expression in live *mcherry-luc@gapdh* gametocytes. Gametocyte stage II, III and IV are shown. Nuclei were stained with Hoechst-33342. All pictures were recorded with standardized exposure/gain times to visualize differences in fluorescence intensity (mCherry 0.7 s; Hoechst 0.136 s; bright field 0.62 s (1x gain)). Bright field (BF), Hoechst (H), mCherry (mC), Merge (M). Scale bar, 7µm.

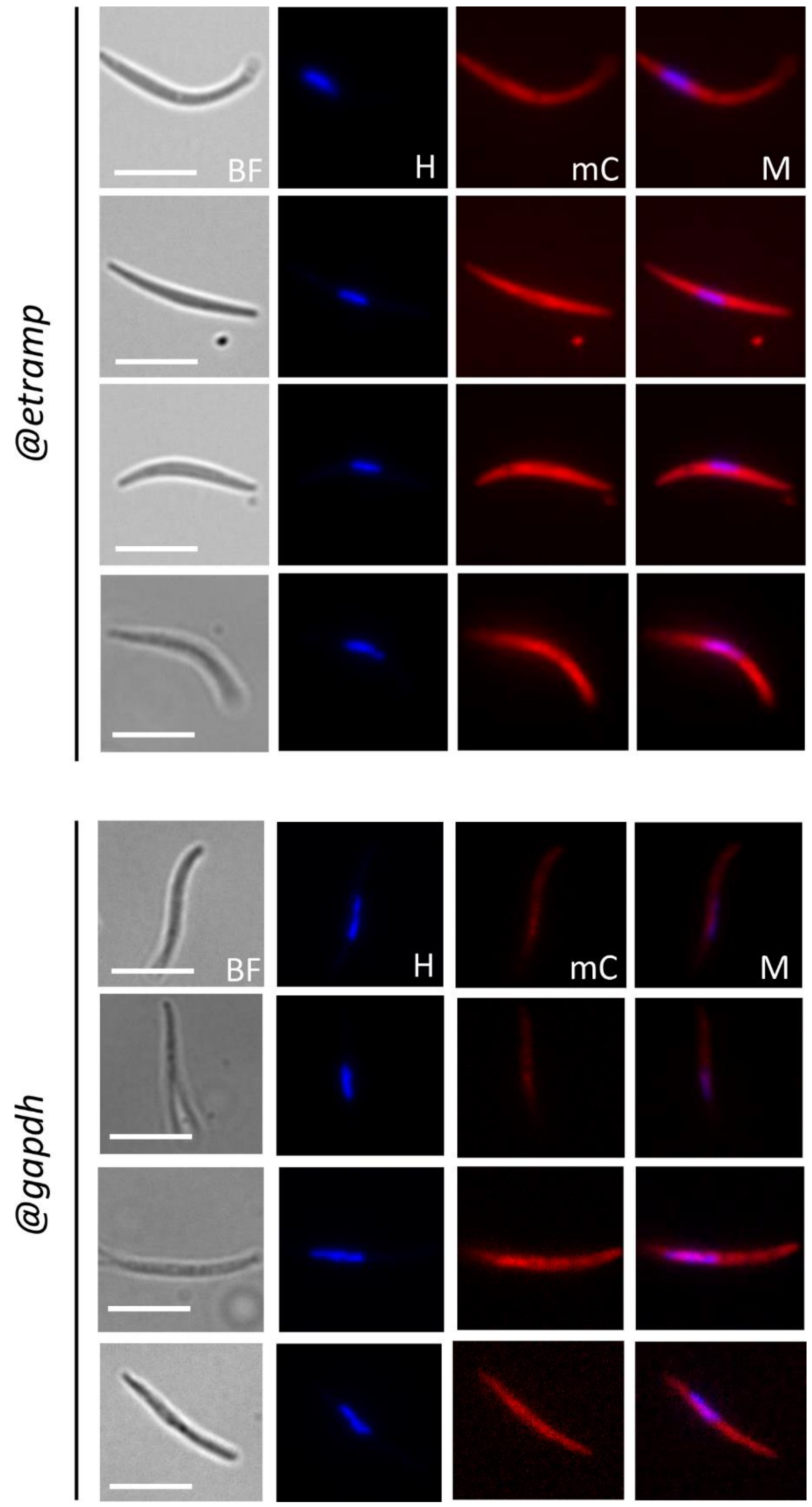

Supplementary Figure 4. **mCherry fluorescence of *mCherry-luc@etramp10.3* and *mCherry-luc@gapdh* salivary glands sporozoites**

Upper panel:: m*Cherry-luc@etramp10.3* salivary gland sporozoites (*@etramp*). Lower panel: *mCherry-luc@gapdh* salivary glands sporozoites (*@gapdh*). Nuclei stained with Hoechst-33342. All pictures were recorded with standardized exposure/gain times; mCherry (red) 0.6s; Hoechst (blue) 0.136 s; bright field 0.62 s (1x gain). Bright field (BF), Hoechst (H), mCherry (mC), Merge (M).Scale bar, 20µm.

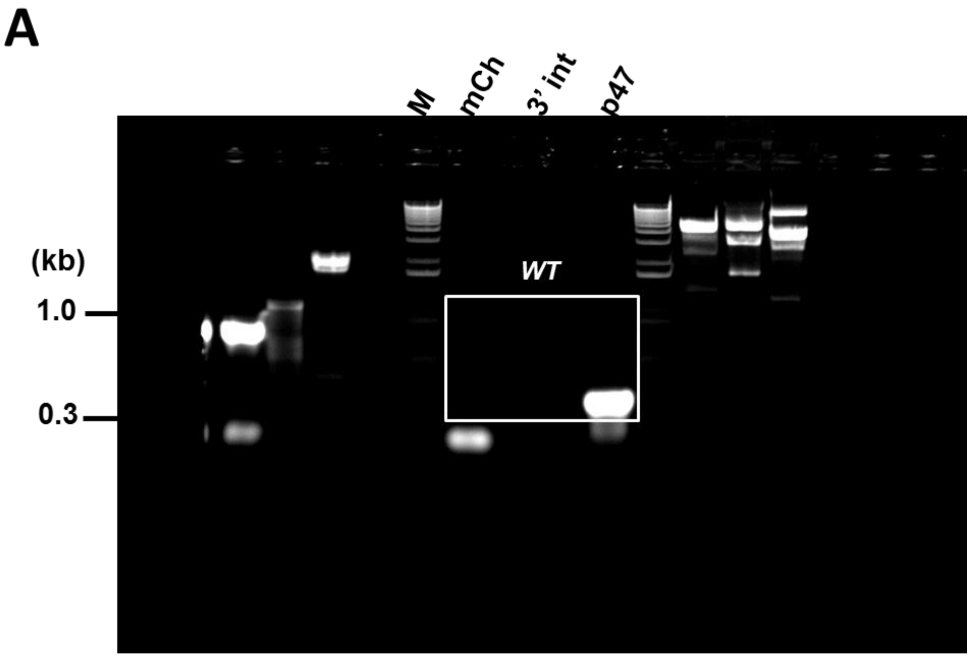

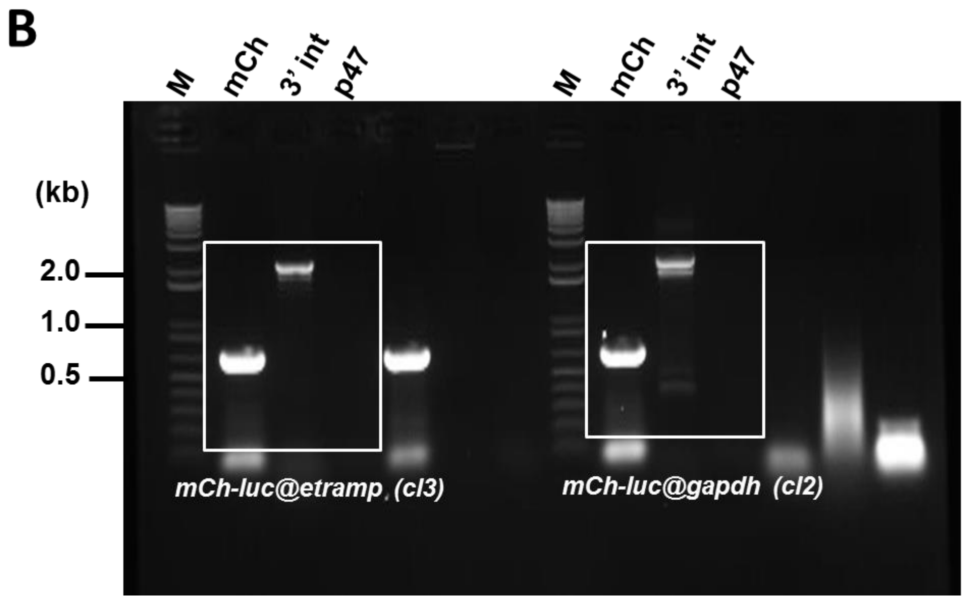

Supplementary Figure 5. **Unprocessed images of PCR analysis.**
The white boxes show the cropped images that correspond to PCR results in Fig.1B (right panel) and Supplementary Fig. S2 (right panel).
(A) PCR results of WT NF54, for both Fig. 1B and Supplementary Fig. S2**.**
(B) PCR results of *mCh-luc@etramp (cl3)* and *mCh-luc@gapdh (cl2)* for Fig. 1B and Supplementary Figure S2. Molecular marker: 1Kb plus ladder (M). DNA fragments were separated in a 0,8% agarose gel.

A

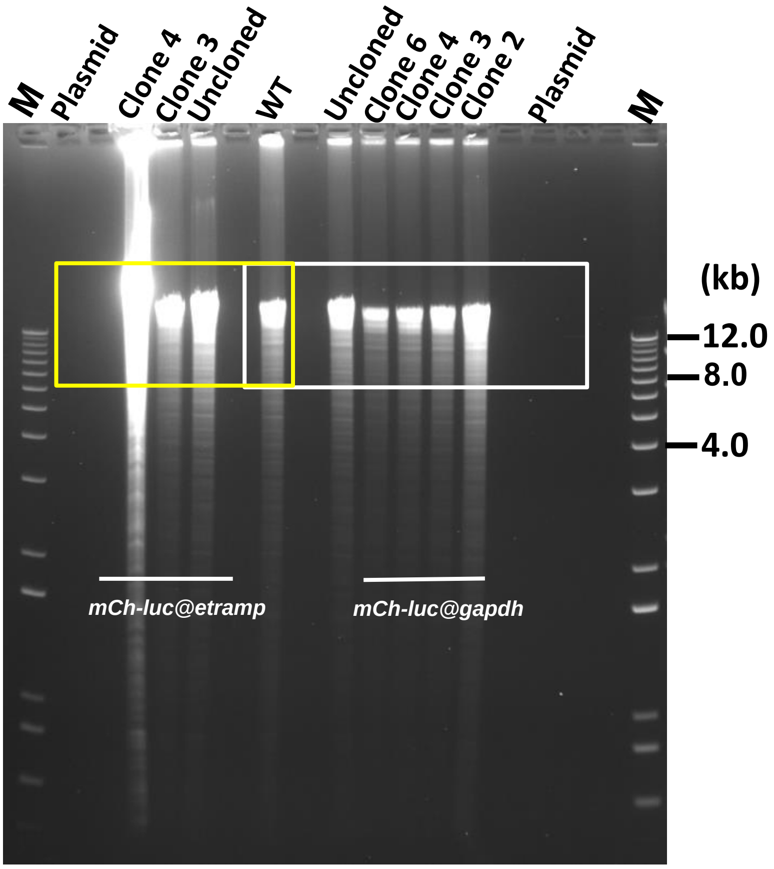
M
Plasmid
Clone 4
Clone 3
Uncloned
WT
Uncloned
Clone 6
Clone 4
Clone 3
Clone 2
Plasmid
M
(kb)
12.0
8.0
4.0
mCh-luc@etramp
mCh-luc@gapdh

B

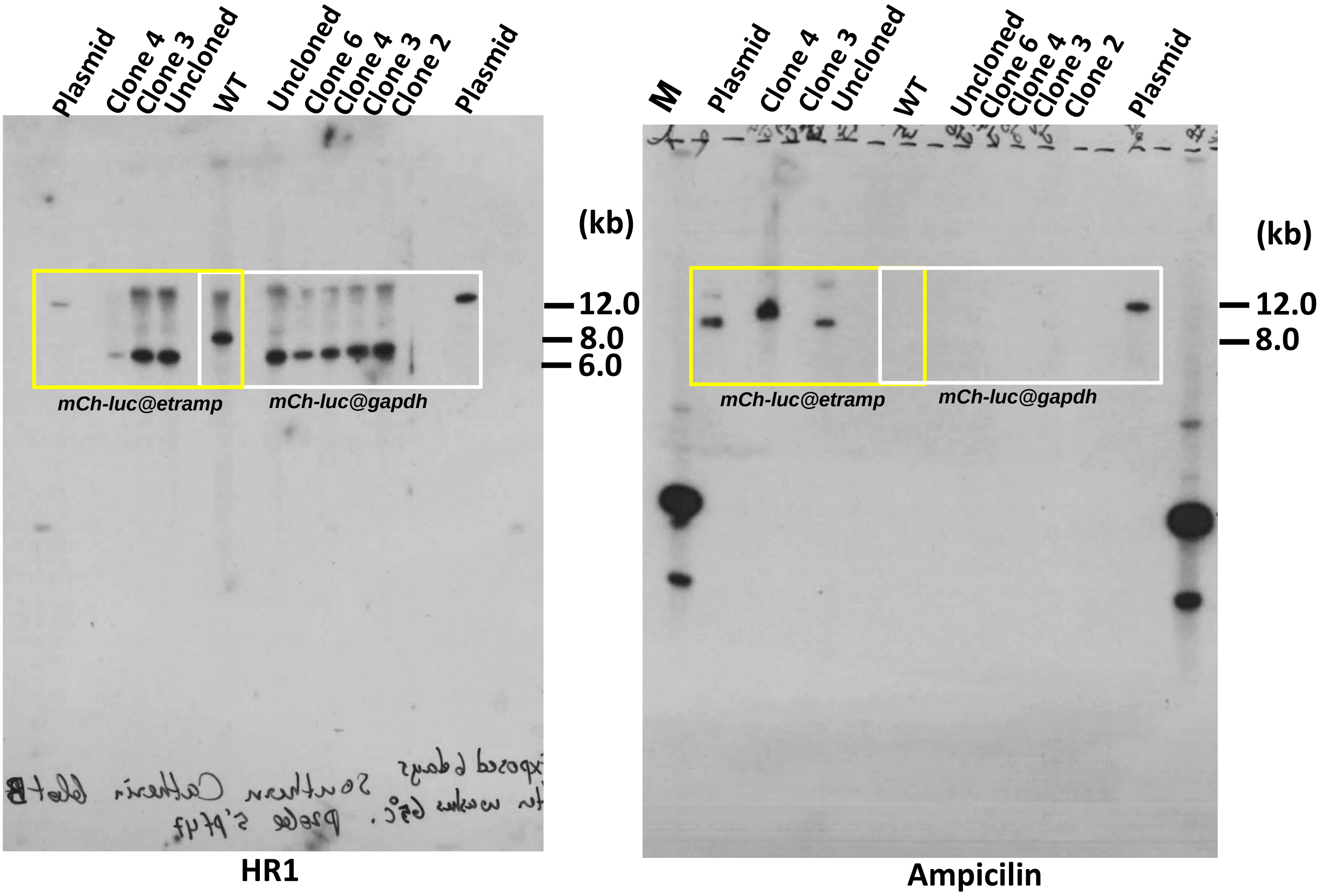
Plasmid
Clone 4
Clone 3
Uncloned
WT
Uncloned
Clone 6
Clone 4
Clone 3
Clone 2
Plasmid
(kb)
12.0
8.0
6.0
mCh-luc@etramp
mCh-luc@gapdh
HR1
M
Plasmid
Clone 4
Clone 3
Uncloned
WT
Uncloned
Clone 6
Clone 4
Clone 3
Clone 2
Plasmid
(kb)
12.0
8.0
mCh-luc@etramp
mCh-luc@gapdh
Ampicilin

Supplementary Figure 6. Unprocessed images of Southern blot analyses. The yellow boxes show the cropped image in Fig.1B (left panel) and white boxes showed the cropped image in supplementary Fig. S2B (left panel).

(A) Gel separated (0,8% agarose gel), restricted DNA (1.5ug per lane) from different clones of *mCh-luc@etramp* and *mCh-luc@gapdh*, WT NF54 and plasmid, stained with ethidium bromide. Molecular marker 1Kb plus ladder (M).

(B) Autoradiograph images after probing restricted DNA (see **A**) with probes HR1 (left panel) and ampicillin (right panel).

Supplementary Table 1. List of primers used in this study

Primer ID	Leiden code	Gene ID	Sequence	Enzymes	Product (bp)	Description
Generation of constructs						
P1	8186	PF3D7_1346800	TAATTAGGCCTGTTGCCGGCGCATACACATAAATATTTGTGTTGTAC	*Stu*I/*Nae*I	794	Forward HR 1 *pfs47*
P2	8187	PF3D7_1346800	TTCCTCCGCGGGATATCCCTCCCACACTCTTGTC	*Sac*II/*EcoRV*		Reverse HR 1 *pfs47*
P3	8122	PF3D7_1346800	TTATTGGGCCCGTCGACGCAATAAATTCATCGTTCAGTG	*Apa*I/*Sal*I		Forward HR 2 *pfs47*
P4	8123	PF3D7_1346800	TCCTTAAGCTTCCCGGG CCACCTTGTTCACAAATACATC	*Hind*III/*Sma*I		Reverse HR 2 pfs47
P5	8126	PF3D7_1346800	TAAGTATATAATATTCAGTTGGCTTAACATTAGTCGTTTTAGAGCTAGAA			Forward gRNA019
P6	8127	PF3D7_1346800	TTCTAGCTCTAAAACGACTAATGTTAAGCCAACTGAATATTATATACTTA			Reverse gRNA019
P7	7919		GTGCCACTTTTTCAAGTTGATAACG			Sequencing gRNA019
P8	5341		GGCATCAGAGCAGATTGTAC			Sequencing gRNA019
P9		PF3D7_1016900	AT**CCGCGG**ATAATTGTCGAAGGTTTACACATAAGGAATG	*Sac*II		Forward *etramp 10.3*
P10		PF3D7_1016900	CCGG**GGTACC**TTTGTCGGAAATCGGATAAGAAGAAAAAAAAATAATATAAAA ATAAAG	KpnI	1703	Reverse *etramp 10.3*
P11	8104	PF3D7_1462800	AACTATTCGCGACCGCGGGCTATGAAAAACATGGGTGTG	*Nru*I/*Sac*II		Forward *gapdh*
P12	8105	PF3D7_1462800	CAATACTCGAGGGTACCGAAAAGAATTAAAAAGCCGAAG	*Xho*I/*Kpn*I	1654	Reverse *gapdh*
Genotyping						
P13	2257		AAAGGTACCTAAAAGAAATATGAGAAC		716	Forward mCherry reporter
P14	2258		AAAAAGCTTTTTCGCCACAGGAGAAAC			Reverse mCherry reporter
P15	6767		TACGTCGCCAGTCAAGTAAC		2188	Forward 3'integration
P16	8297		CATCGAAATGCGTATTAATATGAC			Reverse 3'integration
P17	8428	PF3D7_1346800	AACTATTAAGCTCAACACAATACG		216	Forward Pfs47 ORF
P18	8429	PF3D7_1346800	CTAAATGATATGCGCTGGAATC			Reverse Pfs47 ORF

Supplementary Table 2. Gametocyte, oocyst and sporozoite production in WT and *mCherry-luc@etramp10.3* parasites

Lines	No. of stage V gametocytes[1] range (S.D)	No. of exflagellations[2] range (S.D.)	No. of oocyst[3] range (S.D.)	No of sporozoites (x10^3)[4] range (S.D.)
**WT**<br>*Pf* NF54	**range:**<br>Male: 0.5-0.9 (0.2)<br>Female: 0.9-1.7(0.3)<br>(3 exp.)	**range:**<br>1000-1700 (191)<br>(5 exp.)	**range:**<br>12-60 (18)<br>(5 exp.)	**range:**<br>8-20 (1.5)<br>(3 exp.)
mCh-Luc@etramp<br>line 0064cl3	**range:**<br>Male: 0.4-0.9 (0.3)<br>Female: 0.9-1.8 (0.6)<br>(2 exp.)	**range:**<br>200-1300 (540)<br>(3 exp.)	**range:**<br>20-34 (6.6)<br>(3 exp.)	**range:**<br>8-10 (1.1)<br>(3 exp.)

[1] Percentage stage V male and female gametocytes (per 100 red blood cells) in day 14 cultures.
[2] Number of exflagellating male gametocytes per 10^5 red blood cells at 10-20 min after activation of day 14 gametocyte cultures.
[3] Mean number of oocyst per mosquito at day 9-10 after feeding (10-30 mosquitoes per exp.).
[4] Mean number of salivary gland sporozoites per mosquito at day 21 after feeding (20-30 mosquitoes per exp.)
